# Correlations between Molecular Landscape and Sonographic Image of Different Variants of Papillary Thyroid Carcinoma

**DOI:** 10.3390/jcm8111916

**Published:** 2019-11-08

**Authors:** Andrzej Lewiński, Zbigniew Adamczewski, Arkadiusz Zygmunt, Leszek Markuszewski, Małgorzata Karbownik-Lewińska, Magdalena Stasiak

**Affiliations:** 1 Department of Endocrinology and Metabolic Diseases, Medical University of Lodz, 93-338 Lodz, Poland; zbadam@o2.pl (Z.A.); arkadiusz.zygmunt@umed.lodz.pl (A.Z.); 2 Department of Endocrinology and Metabolic Diseases, Polish Mother’s Memorial Hospital—Research Institute, 93-338 Lodz; Poland; leszekmarkuszewski@gmail.com (L.M.); mkarbownik@hotmail.com (M.K.-L.); mstasiak33@gmail.com (M.S.); 3 Chair and Department of Oncological Endocrinology, Medical University of Lodz, 90-752 Lodz, Poland

**Keywords:** papillary thyroid carcinoma, ultrasound, sonographic features, *BRAF^V600E^* mutations, *RAS* mutation, genetic drivers, classic variant, follicular variant

## Abstract

Papillary thyroid carcinoma (PTC), the most common thyroid cancer, is predominantly driven by mutations in *BRAF* (primarily p. *V600E*) and *RAS* oncogenes. Ultrasound (US) examination provides significant diagnostic data in the management of thyroid nodules, as many sonographic features of thyroid lesions are correlated with the potential risk of thyroid carcinoma. The aim of the study was to analyze the current literature in regard to the potential associations between genetic landscape and sonographic features of PTC. Based on the current literature, sonographic features of PTCs correlate with their molecular drivers, particularly between tumors harboring *BRAF^V600E^* versus activating *RAS* mutations, although many of these findings appear to be dependent on the tumor variant. Suspicious US findings, such as hypoechogenicity, spiculated/microlobulated margins, non-parallel orientation/taller-than-wide shape, and the presence of microcalcifications, are typical for PTC positive for *BRAF^V600E^* mutations. On the contrary, tumors with *RAS* mutations are most frequently hypo- or isoechoic and ovoid-to-round in shape, with smooth margins and without calcifications. There are also some US features typical for PTCs harboring other mutations, including *BRAF^K601E^*, *RET/PTC* rearrangements, *PAX8-PPARγ*, *CTNNB1*, and *APC*. However, further research is necessary, as some rare PTC variants still cannot be reliably analyzed due to the scarce published data.

## 1. Introduction

Papillary thyroid carcinoma (PTC) is the most common thyroid cancer, accounting for over 90% of all thyroid malignancies. Several mutations comprise the underlying molecular etiology of PTC, including *BRAF* (predominantly p. *V600E*) and activating *RAS* mutations. Less frequent drivers include other non-*V600E BRAF* mutations, *APC* and *CTNNB1* mutations, as well as *NTRK*, *ALK*, and *BRAF* fusions [1].

Ultrasound (US) examination is a precise diagnostic tool in the management of thyroid nodules as many sonographic features of thyroid lesions provide information about the potential risk of thyroid carcinoma. Moreover, sheer wave sonoelastography was demonstrated to provide significant additional data facilitating the assessment of the risk of malignancy [2]. Many US-based classifications of thyroid nodules have been formulated to facilitate the selection of lesions for fine needle aspiration biopsy (FNAB) [3,4,5,6,7,8]. The majority of the classifications are consistent in regard to the most suspicious features, including hypoechogenicity, blurred margins, microcalcifications, taller-than-wide shape, and—undoubtedly—the presence of metastatic lymph nodes. 

It seems reasonable to believe that the presence of a specific mutation leading to PTC may affect the ultrasound image of the lesion. Recently, we demonstrated that genetic drivers can significantly affect the US pattern of thyroid lesions in subacute thyroiditis [9]. It can be expected that such an association would be even more pronounced in thyroid malignant lesions. Several authors have attempted to analyze the correlation between the molecular landscape of thyroid carcinoma and the ultrasound image. Unfortunately, these reports are scarce and significant discrepancies between the authors’ observations are often present.

The aim of this review is to answer the question whether—on the basis of the most recent literature—there is a relationship between the genetic drivers of PTCs and their ultrasound image. A study selection flowchart is available in the Supplementary Material—Figure S1.

## 2. Thyroid Tumor Classifications

### 2.1. New Aspects of Pathological Classifications of Thyroid Carcinoma

The most recent improvements in thyroid nodule classifications began in March 2015, when the Endocrine Pathology Society reclassified encapsulated follicular variant of PTC (EFV PTC) or clearly delimited non-invasive follicular variants of papillary thyroid carcinoma under the proposed nomenclature “non-invasive follicular thyroid neoplasm with papillary-like nuclear features” (NIFTP) [10,11]. This reclassification was approved in 2017 by the American Thyroid Association and was incorporated into the 2017 WHO classification of tumors of the thyroid gland under a new class of diagnoses “other encapsulated follicular-patterned thyroid tumors” [3,11]. 

The new WHO classification schema also introduced two additional diagnoses under this new category: A follicular tumor of uncertain malignant potential (FT-UMP) and a well-differentiated tumor of uncertain malignant potential (WDT-UMP), both of which were borderline between follicular adenoma and carcinoma or follicular adenoma and a follicular variant of papillary carcinoma. In these new entities, nuclear features of PTC as well as lack of capsular or vascular invasion are not so clearly expressed as in NIFTP [11].

Importantly, a new classification of PTC variants was provided, with the following variants being included: (1) Conventional/classic variant; (2) papillary microcarcinoma; (3) encapsulated PTC—applicable to fully encapsulated classic PTC; (4) follicular variant (FV)—invasive PTC with an exclusively or almost exclusively follicular pattern, and no developed papillae; (5) diffuse sclerosing variant; (6) tall cell variant; (7) columnar cell variant; (8) cribriform-morular variant; (9) hobnail variant—new entity; (10) PTC with fibromatosis/fascitis-like; (11) solid/trabecular variant; (12) oncocytic variant; (13) spindle cell variant and giant cell carcinoma; (14) clear cell variant; (15) Warthin-like variant; and (16) combined papillary and medullary carcinoma [11]. The above classification of thyroid tumors is extremely detailed and includes 16 variants of papillary thyroid carcinoma, 5 of which are—in contrast to the others—characterized by a particularly poor prognosis (aggressive biological behavior). These are tall cell variant (being definitely the most common among the aggressive variants), diffuse sclerosing variant, columnar cell variant, cribriform-morular variant, and hobnail variant (new entity) [11]. Cribriform-morular variant is considered by many authors as more favorable [12], but in most cases, its course is very aggressive despite the “benign-like” sonographic pattern.

### 2.2. Sonographic Classifications of Thyroid Nodules

In recent years, several classifications of thyroid nodules based on the presence of US high-risk features were introduced [3,4,5,6,7,8]. The aim of these classifications is to limit the number of unnecessary FNAB and, at the same time, to facilitate the proper qualification to FNAB of all lesions with the potential US risk of malignancy. The most commonly used classifications are prepared by the American Thyroid Association (ATA) [3], American Association of Clinical Endocrinologists (AACE/ACE/AME) [13], American College of Radiology (ACR) [14], European Thyroid Association (ETA) [3], and Korean Society of Thyroid Radiology [5].

ATA classification distinguishes five groups of US risk—benign, very low suspicion, low suspicion, intermediate suspicion, and high suspicion [3]. The last group includes nodules that are solid and hypoechoic, or partially cystic with a solid hypoechoic component, with one or more of the following features: Irregular margins, microcalcification, taller-than-wide shape, rim calcification with small extrusive soft tissue component, and evidence of extrathyroidal extension. Hypoechoic solid nodules without any of these additional features are considered as part of the intermediate suspicious group [3]. Similar US features allow classification of the lesion as high risk in the EU-TIRADS classification (EU-TIRADS 5 category) [4]. All classifications consider smooth margins and isoechogenicity as unsuspicious US features; however, some aggressive variants of PTC may have such a sonographic pattern. Many authors believe that point classifications better assess the real sonographic risk of the nodule. In 2013, we proposed such classification, giving 3 points for nodule augmentation and for suspicious lymph nodes; 1 point for hypoechogenicity, microcalcifications, suspicious shape, and vascularization; and 0.5 points for blurred margins, size, presence of halo, and echostructure [6,7,8]. The total sum of 7 points or more was considered as a high-risk sonographic pattern [6,7,8]. The same number of points for a highly suspicious nodule was proposed by Tessler et al. [14] in the point classification developed by ACR (ACR-TIRADS). This classification provides recommendation for FNAB for five risk classes selected on the basis of the total number of points (TR level 1–5) [14]. Recently, integration of sonoelastography into the TIRADS classification was demonstrated to increase the value risk of predicted malignancy, with a consequently different approach to further clinical investigation and management [15].

## 3. Genetic Drivers of Thyroid Carcinoma 

Initiating mutations or gene fusions in the thyroid carcinoma include: *BRAF* in PTC, *N-K-H-RAS* in PTC and/or follicular thyroid carcinoma (FTC), *RET/PTC* in PTC, and *PAX8/PPARγ* in FTC [16]. *RET/PTC1*, *RET/PTC3*, and *RET/PTC4* are formed as a result of intrachromosomal rearrangements (paracentric inversions within the long arm of chromosome 10). Several other *RET/PTC* rearrangements result from inter-chromosomal translocations [17].

The prevalence of mutations according to the thyroid tumor type was demonstrated as follows: PTC: *BRAF* in 40% to 45%, *RET/PTC* in 6% to 30%, telomerase reverse transcriptase (*TERT*) promoter in up to 23.5%, *RAS* in 10% to 20%, and *TRK* in <5%; FTC: *RAS* in 40% to 50%, *PAX8-PPARγ* in 10% to 35%, *PIK3CA* in <10%, and *PTEN* in <10%; poorly differentiated carcinoma: *RAS* in 25% to 30%; *CTNNB1* in 10% to 20%, *TP53* in 20% to 30%, and *BRAF* in 10% to 15%; familial forms of medullary carcinoma (MTC): *RET* in >95%; and sporadic MTC: *RET* in 40% to 50% [16,18,19].

Some of the mutations can serve as molecular markers of the aggressiveness of differentiated thyroid carcinoma (DTC). Such markers include the presence of >1 driver mutation, and *PIK3CA*, *p53*, *CTNNB1*, and *TERT* promoter mutations [18,20].

## 4. Genetic Drivers and Sonographic Pattern of Different Variants of PTC

### 4.1. BRAF Mutations 

*BRAF* mutations have been reported in classic, Hürthle, tall cell, columnar cell, hobnail, Warthin, and diffuse sclerosing variants. *BRAF^V600E^* is a common mutation characteristic for PTC displaying a papillary or mixed follicular-papillary growth pattern [21]. Wang et al. [22] demonstrated via multivariate analysis that poorly defined margins and microcalcification were independent predictors of *BRAF^V600E^* mutation. Lee et al. [23] found that *BRAF^V600E^* mutation was associated with the following US features: Solid composition, marked hypoechogenicity, irregular margin, taller-than-wide shape, and the presence of microcalcifications. Similarly, Khadra et al. [24] observed that hypoechogenicity, intra-nodular calcification, and irregular nodular margins were the most predictive features of *BRAF^V600E^* positivity. Unfortunately, they did not analyze the tumor shape. Additionally, they observed that *BRAF^V600E^*-positive PTCs were associated with a higher rate of lymph node metastasis and the rate of extrathyroidal extension [24]. Very recently, Colombo et al. also demonstrated a correlation between *BRAF^V600E^* mutation and extrathyroidal invasion, as well as US features of the higher ATA risk category of PTC [18]. In the analysis by Rossi et al. [25], *BRAF^V600E^* mutation was associated with hypoechogenicity, microcalcifications, and a nodule diameter <1 cm. Kabaker et al. [26] observed that *BRAF^V600E^* positivity was associated with such suspicious US findings as a taller-than-wide shape, ill-defined margins, hypoechogenicity, micro/macrocalcifications, and an absent halo. On the contrary, *BRAF^V600E^* mutations were not associated with non-cystic composition. The authors stated that *BRAF* mutation can be predicted with a positive predictive value of 82% when at least three of the above-mentioned suspicious US features are present [26]. A taller-than-wide shape was proven to be a useful US feature for predicting thyroid malignancy, especially when analyzed in both transverse and/or longitudinal planes [27]. Those observations were not dependent on the PTC variant, as the authors did not analyze correlations with PTC variants [22,23,24,25,26,27].

The classic variant is the most common PTC variant (32–49%), and the *BRAF^V600E^* mutation is the most frequent (90%) genetic driver of this variant [28,29,30]. A typical US pattern for this PTC subtype includes hypoechogenicity, spiculated/microlobulated margins, microcalcifications, non-parallel orientation, and mixed-type non-increased vascularity [31]. 

In the Hürthle cell variant, *BRAF^V600E^* was detected in tumors displaying a papillary or a mixed growth pattern but not in those with a follicular pattern [21]. In US, the lesion can be hypo- or isoechoic with smooth or spiculated/microlobulated margins. Microcalcifications are often absent, but macrocalcifications are found in 20% of tumors. A non-parallel orientation is very characteristic, and vascularity is mixed in most patients and increased in half of the cases [31].

The incidence of tall cell variant, which is also frequently (over 92%) associated with *BRAF^V600E^* mutations, is reported as 4% to 17% of PTCs [12]. Ultrasounds reveal malignant features typical for *BRAF^V600E^* mutation, which include a solid structure, hypoechogenicity, with or without microcalcifications, a spiculated/microlobulated margin, and non-parallel orientation with frequent nodal metastass [12,31].

The very rare columnar cell variant also presents typical malignant features, and *BRAF^V600E^* mutation occurs in one-third of these PTCs [32]. Encapsulated tumors of this variant exhibit a US pattern of circumscribed hypoechoic nodules. Such cases are associated with better prognosis. Microcalcifications may be present but they are not typical. More aggressive tumors are larger hypoechoic ones, with microlobulated margins, often associated with visible extrathyroidal invasion and lymph node metastases [12,32]. Interestingly, *BRAF^V600E^* mutation is frequent in these more aggressive tumors while the encapsulated ones are often *BRAF^V600E^* negative [32]. Chen et al. [32] indicated that altered *BRAF^V600E^* is an adverse prognostic factor in the columnar cell variant due to its association with unfavorable clinicopathological characteristics. Older patient age, male gender, and distant metastasis were highly prevalent features for these *BRAF*-positive tumors [32]. The rare aggressive hobnail variant was reported to be associated with *BRAF^V600E^* mutation in 58% of cases. [33]. In US, these lesions are usually described as microlobulated hypoechoic nodules with microcalcifications and multiple lymph node metastases [12,33]. Tumors are often multifocal [33].

The Warthin-like variant (WV) is associated with Hashimoto’s thyroiditis in 93% to 100% of all cases [12]. Typically, cells with nuclear features of papillary carcinoma line the papillary structures, with dense lymphocytic infiltration in the stalks. In this variant, *BRAF^V600E^* mutations are less frequent than in the classic variant (65% vs. 84%) [12], but *BRAF* mutation is still the most common genetic driver. The most common US features of WV PTCs are a solid composition, hypoechogenicity, and taller-than-wide shape [34,35]. The US pattern of nodules harboring *BRAF^V600E^* mutations may differ depending on the presence of Hashimoto’s thyroiditis. Microcalcifications were observed as typical for *BRAF^V600E^*-positive nodules without Hashimoto’s thyroiditis while a solid nodule structure was more common in *BRAF^V600E^*-positive nodules with Hashimoto’s thyroiditis [36].

Papillary thyroid microcarcinoma (PTMC) constitutes approximately 30% of all PTC cases, and it is also typically associated with *BRAF* mutation, with a US pattern typical for these mutations [28,30].

*BRAF^V600E^* mutations are rare (24%) in the diffuse sclerosing variant (DSV) [37], but their presence significantly influences the US pattern of DSV. The challenges related to dependencies between the molecular landscape and the sonographic pattern of this subtype will be discussed further below.

On the basis of the presented literature review, PTCs harboring *BRAF^V600E^* mutations frequently demonstrate typical US high-risk features, including hypoechogenicity, microcalcifications, irregular and/or ill-defined margin, taller-than-wide shape, and extrathyroidal invasion. 

**Figure 1:**
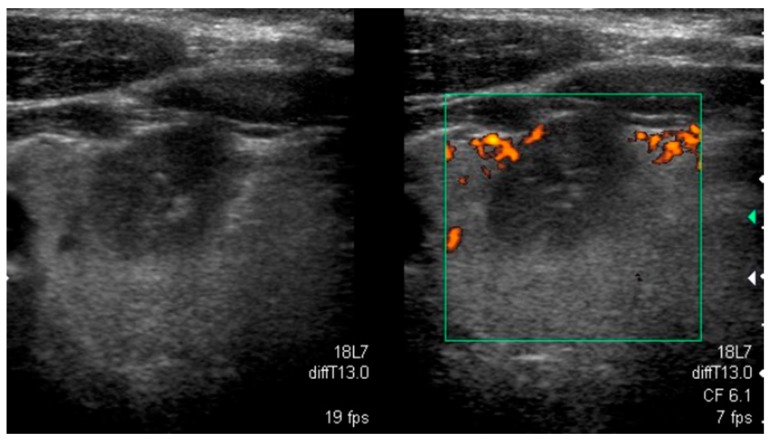
Typical sonographic pattern of a *BRAF^V600E^*-positive papillary thyroid carcinoma hypoechoic lesion with poorly defined irregular margins and microcalcifications.

A sonographic pattern typical for PTCs bearing the *BRAF^V600E^* mutation is presented in Figure 1.

The correlations between the presence of *BRAF^V600E^* mutations and sonographic findings in PTC are widely described, although there are also a few reports in which such associations were not observed [29,38]. The authors of these reports analyzed the US patterns of *BRAF^V600E^*-positive and –negative tumors without any assessment of the PTC variants. The authors claimed not to observe an association with the US pattern, although correlations between the presence of *BRAF^V600E^* mutations and features, such as a larger tumor size, extrathyroidal extension, and lymph node metastases, were described [29].

*BRAF^K601E^* mutations are present in most cases of the infiltrative follicular variant (FV) [12]. In US, these nodules are often hypoechoic, with spiculated/microlobulated margins and microcalcifications [31,39]. The differences in the US pattern of FV PTC depending on genetic drivers are discussed further below.

### 4.2. RET/PTC Rearrangements

**Figure 2:**
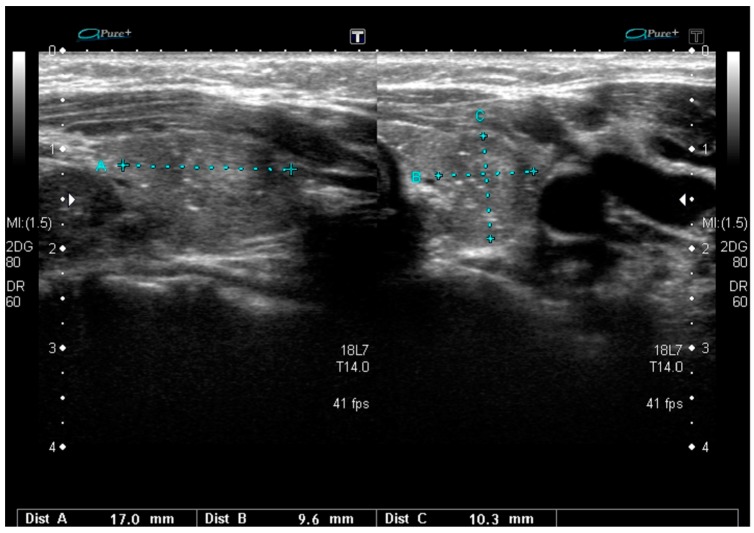
Sonographic pattern typical for the *RET/PTC3*-dependent diffuse sclerosing variant of PTC with a poorly defined area with heterogeneous parenchyma and scattered microcalcifications.

*RET/PTC* rearrangements are the second most common genetic alteration in PTC, mainly being a genetic driver of the classic variant. More rarely, it can be associated with other variants of PTC, especially with the diffuse sclerosing variant (DSV) [19,40]. The two most common types of rearrangements in PTC are paracentric inversions, *RET/PTC1* and *RET/PTC3*. The prevalence of *RET/PTC* rearrangements in PTC is 6% to 30%, and they occur more commonly after radiation exposure and/or in children [18,19]. Data on the correlation between this genetic alteration and US patterns of PTC are very scarce and frequently inconsistent [12,19,25,37]. Isoechogenicity was described as being more common than hypoechogenicity in nodules with *RET/PTC3* rearrangement [25]. Microcalcifications are present in the majority of cases [19,25]. Additionally, small-sized noduled and lymph node metastases are also typical in US [19]. *RET/PTC* rearrangement is the most common genetic feature in DSV [12,19]. The diffuse sclerosing variant frequently occurs in young women [12]. Despite having a similar genetic landscape, the US patterns of DSVs and classic variants are entirely different. Characteristic features of DSV include diffuse involvement of one or both thyroid lobes (frequently, no single separated tumor nodule is present), with heterogeneous parenchyma, ill-defined margins, solid hypoechoic nodules (if it is possible to separate any nodule), and scattered microcalcifications within or without the defined nodule (a “snowstorm” pattern). Additionally, lateral nodal involvement with typical nodal microcalcifications is often found [12]. This variant may sometimes resemble chronic thyroiditis. In cytology, extensive squamous metaplasia, numerous psammoma bodies, dense lymphocytic infiltration, and stromal fibrosis are typically described. The US pattern of DSV harboring *RET/PTC* rearrangements seems to be different in *RET/PTC1*- and *RET/PTC3*-positive cancers. Typical diffuse involvement with scattered microcalcifications without any visible nodule is much more frequent in *RET/PTC3*-driven tumors. The coexistence of DSV and Hashimoto’s thyroiditis is characteristic for *RET/PTC1* mutations. In these last cases, microcalcifications are less frequent than in *RET/PTC3* tumors [37]. *BRAF^V600E^* mutations are rare (24%) in DSVs [37] and the sonographic pattern of such tumors is quite similar to those that harbor *RET/PTC1* fusions and are significantly different from those with *RET/PTC3* rearrangement. In US, *BRAF^V600E^*-positive DSVs are more similar to other PTCs that harbor *BRAF^V600E^* than a typical DSV pattern, as the nodules are usually clearly visible and diffuse microcalcifications occur in only 33% of cases [37]. A sonographic pattern typical for PTCs harboring *RET/PTC3* rearrangement are presented in Figure 2.

### 4.3. RAS and PAX8-PPARγ Mutations

**Figure 3:**
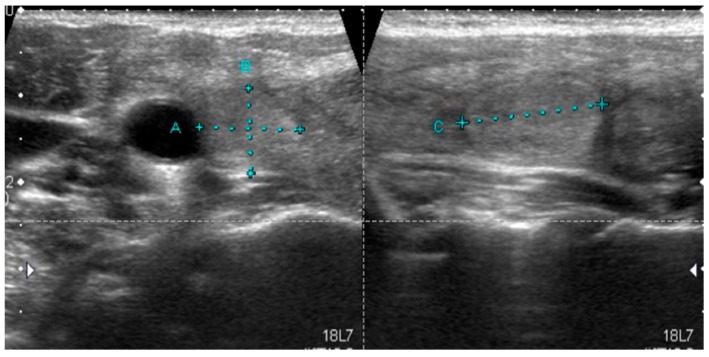
Typical sonographic pattern of *RAS*-positive NIFTP, normoechoic lesion with well-defined margins and without calcifications.

*RAS* mutations are typical for follicular tumors (including adenomas and carcinomas), but they also occur in the follicular variant (FV) of PTC. The follicular variant is the second most common type of PTC after the classic variant [19,40]. Nuclear features are typical for PTC, but the growth pattern is follicular. Based on the molecular alterations and biological behavior, FV tumors were divided into two main subtypes of FV PTC: Infiltrative and encapsulated with invasion [40]. As it has already been mentioned, the lesions previously classified as the follicular variant of PTC (including EFVPTC) now include only those lesions with angiolymphatic or capsular invasion, or a wandering/infiltrative border. Well-demarcated or encapsulated lesions without invasion belong to the newly described entity NIFTP [11]. NIFTP is a tumor with a very good prognosis and rather benign behavior. The extent of surgical procedures can be limited in this case, although surgery is required. NIFTPs usually harbor activating *RAS* mutation (30–59%), including *N-RAS*, *H-RAS*, and, less commonly, *K-RAS* mutations [19,40,41,42,43,44]. A lack of *BRAF^V600E^* mutation, detected by molecular assays or immunohistochemistry, and lack of *BRAF^V600E^*-like mutations or other high-risk mutations (*TERT*, *TP53*) were included into the 2018 revised diagnostic criteria for NIFTP as secondary criteria [45]. Rosario and Mourão [46] analyzed a series of papers that reported the presence of *BRAF^V600E^* in NIFTP and demonstrated that, initially, the tumors were probably inappropriately classified as NIFTP, but in a further assessment, the presence of papillae and/or even capsular invasion was observed. *BRAF^K601E^* mutation was found in a subset of NIFTP cases (4%) [47,48], but this group may require a similar reassessment. Although NIFTPs predominantly bear *RAS* mutations, tumors with other genetic alterations, such as *PPARγ* and *THADA* mutations, were also described [44,49]. NIFTP tumors did not present malignant US features. They are frequently isoechoic or even hyperechoic, with well-defined margins and no microcalcifications [46,50,51]. Rosario et al. [52] demonstrated that sonographic images of NIFTP thyroid nodules were classified according to ACR as TIRADS 5 only in 3.5%. More frequently, they corresponded to TIRADS 4 (67.8%) or TIRADS 3 (28.5%) [52]. Similarly, only a small percentage of NIFTP lesions were classified as ATA high-risk lesions while 66% of them corresponded to the low-risk category [53]. A typical sonographic pattern of NIFTP is presented in Figure 3.

**Figure 4:**
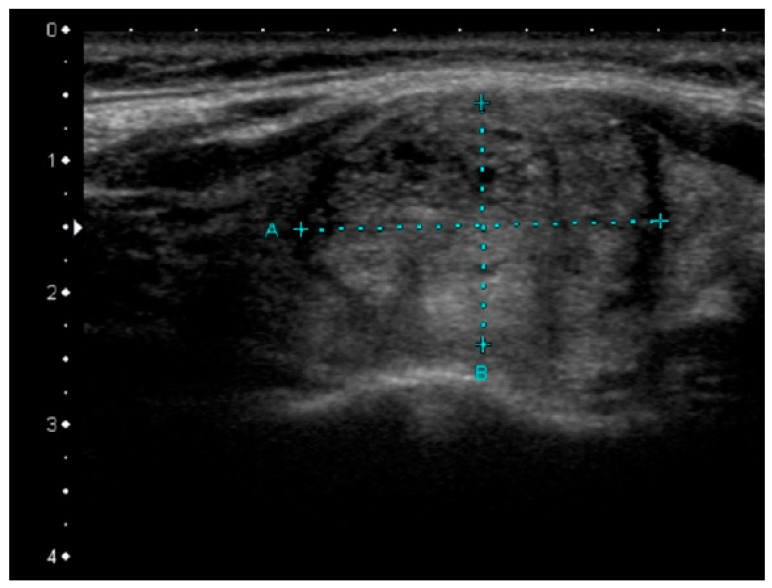
Sonographic pattern of *RAS*-positive papillary thyroid carcinima, slightly hypoechoic, heterogeneous lesion with rather well-defined margins.

According to the 2017 WHO classification, two subtypes of FV PTC are distinguished: Invasive encapsulated FV PTC (i.e., the encapsulated FV with capsular or vascular invasion), and infiltrative FV, which is not encapsulated [11]. Invasive EFV PTC is associated with *RAS* mutation or *PAX8-PPARγ* and it constitutes 4% of all PTCs. Infiltrative FV accounts for 6% of all PTCs and is mainly caused by *BRAF* mutation, usually *BRAF^K601E^*, or, less often, *RAS* mutation [12,21,28,40]. *BRAF* mutations are therefore typical for the more aggressive infiltrative FV PTC while *RAS* mutations are often found in the less aggressive invasive EFV or the above-described NIFTP. US features of FV can suggest the tumor behavior and molecular landscape, as it can have typical PTC-like US patterns with hypoechoic lesions with irregular blurred margins and, sometimes, microcalcifications or rather follicular neoplasm-like patterns, without these markers of potential malignancy. Infiltrative FV is frequently a hypoechoic lesion, with spiculated/microlobulated margins. Microcalcifications can be present in about 20% of the cases. Nodules are often ovoid to round in shape, with a non-parallel orientation and mixed vascularization. The presence of microcalcifications is less common in the FV than in the classic variant of PTC (41.2% vs. 17.6%) [31,39]. Invasive encapsulated FV is typically a hypoechoic lesion with rather smooth margins and no calcifications. Nodule shape, orientation, and vascularity are similar to the infiltrative type of FV [31,39]. Similar observations concerning more benign US features of nodules harboring *RAS* mutations were presented by Rossi et al. [25], who demonstrated that most of them were isoechoic. Nodules bearing *RAS* mutations were also larger than nodules with *BRAF^V600E^* mutations (>1 cm) [25]. Interestingly, de Napoli et al. [54] observed microcalcifications and irregular margins in nodules with *N-RAS* mutation. US examination is a very good prognostic tool in examining follicular-patterned lesions with papillary nuclear features as the infiltrative FV PTC may be differentiated from the benign NIFTP on the basis of the US pattern. However, US-based discrimination between invasive EFVs and NIFTPs is rather impossible in clinical practice [12,55]. The presence of *RAS* mutations is usually associated with less aggressive forms of PTC and a more benign US image (Figure 4). 

However, follicular adenomas and carcinomas [56], and even poorly differentiated [57] and anaplastic [58] thyroid carcinomas, can bear *RAS* mutations. In such cases, the US findings are significantly different from those of NIFTP or invasive encapsulated FV of PTC. The differences in the sonographic pattern of thyroid follicular adenoma and carcinoma are well described, despite the fact that both of them harbor *RAS* mutations [56]. Thus, although PTCs with *RAS* mutations often have more benign US patterns than those with *BRAF* mutations, the presence of *RAS* mutations cannot be determined by any particular sonographic findings. In *RAS*-positive highly aggressive thyroid carcinomas, the US pattern is commonly very suspicious, definitely belonging to the ATA high-risk group [3].

### 4.4. TERT Promoter Mutations

The frequency of the *TERT* promoter mutation is reported as 21%, 12%, and 9% in the classic, hobnail, and cribriform-morular variant (CMV) of PTC, respectively [18,33,59]. The presence of the *TERT* promoter mutation is strongly associated with older age, less-differentiated PTC, and a high risk of recurrence. *TERT* promoter mutations were suggested to be useful surrogate markers for high-risk PTC [60]. The US pattern is highly suspicious in all PTC variants harboring *TERT* promoter mutations. Tumors are typically multifocal, microlobulated, and hypoechoic with microcalcifications, and multiple lymph node metastases are frequently present [12,33]. Kim et al. [60] recently reported that a non-parallel orientation and microlobulated margin of the nodule were independent sonographic findings for predicting *TERT* promoter-mutated PTC in patients older than 50 years. 

### 4.5. Other Mutations in Different Variants of PTC

Several different gene mutations, rarely occurring in PTCs, were described in uncommon variants, such as the *DICER1* mutation in a solid/trabecular variant [61]. The solid/trabecular variant of PTC is very rare, and constitutes about 3% of all PTCs. It occurs more commonly in children and young adults, especially in patients with a medical history of radiation. Histologically, this variant should be distinguished from poorly differentiated thyroid carcinomas, as they demonstrate similar solid, insular, and trabecular growth patterns. In US, these tumors usually have typical malignant features (i.e., hypoechogenicity, microcalcifications, irregular margins), but well-defined margins were also occasionally described [12]. Extrathyroidal extension and lymph node involvement are typically observed in US. A single case of *BRAF^VK600-1E^* mutation was also described in this variant of PTC [62].

Several mutations were described in the exceptionally uncommon cribriform-morular variant (CMV)—a variant of PTC that is usually associated with autosomal dominant inherited familial adenomatous polyposis (FAP). Sporadic isolated cases are very rare [63,64]. Tumors are often multiple. In patients with FAP, CMV PTC may occur first, before colonic manifestations are present. These tumors are often associated with mutations of *CTNNB1*, *APC* [65,66,67]. They have sonographic “benign-like” features, although their biological behavior can be very different, and is quite frequently very aggressive. In US, the tumor is frequently solid, oval to round, circumscribed, heterogeneous, and hypoechoic, without a hypoechoic halo or calcifications [12,67,68,69]. Histologically, CMV is characterized by the papillary growth of tall columnar cells, a cribriform pattern, lack of colloids, and the presence of spindle cells, squamoid morules, and nuclear clearing. β-catenin immunostaining is a hallmark of CMV PTC, both in sporadic cases and in cases associated with FAP [67,68]. As it was indicated above, CMV may be associated with *TERT* promoter mutations [59]. Several other different gene mutations were reported in CMV PTC, including *K-RAS* mutation [70], *RET/PTC* rearrangement, [71], and *PIK3CA* [72]. Unfortunately, no data is available on potential differences between CMVs harboring each of these mutations.

Associations between the genetic landscape and US features of PTC may be observed only for the most common mutations, due to the lack of published data in the cases of rare genetic alterations. Thus, we summarized the reports that provide the largest number of cases and the most detailed description of both US features and molecular findings. The observed correlations between molecular and sonographic aspects of PTC are presented in Table 1.

## 5. Conclusions

On the basis of the current literature data, sonographic features of PTCs correlate with their molecular drivers, particularly between tumors harboring *BRAF^V600E^* versus activating *RAS* mutations, although many of these findings appear to be dependent on the tumor variant. Suspicious US findings, such as hypoechogenicity, spiculated/microlobulated margins, non-parallel orientation, taller-than-wide shape, and the presence of microcalcifications, are typical for PTC harboring *BRAF^V600E^* mutations. On the contrary, tumors with *RAS* mutations are most frequently hypo- or isoechoic, and ovoid to round in shape, with smooth margins and without calcifications. There are also some US features typical for PTCs harboring other mutations, including *BRAF^K601E^, RET/PTC* rearrangement, *PAX8-PPARγ*, *CTNNB1*, and *APC*. However, further research is necessary, as some rare PTC variants still cannot be reliably analyzed due to the scarce literature, and some authors question even the widely described correlations between *BRAF^V600E^* and the typical suspicious US pattern.

## Figures and Tables

**Table 1:** Correlations between genetic drivers and sonographic findings in PTC, with respect to the specific PTC variants.

Mutation	Tumor US Features and References	PTC Variant
*BRAF^V600E^*	solid structure [12,23,31,34,35] hypoechogenicity [12,23,24,25,26,31,34,35] microcalcifications [12,22,23,24,25,26,31,33] macrocalcifications [31] taller-then-wide shape [23,29,34,35]/non-parallel orientations [31] spiculated/microlobulated/irregular margin [12,23,24,31] absent halo [26] ill-defined margin [22,26] lymph node metastases [12,24,33] extrathyroidal extension [12,24] mixed type vascularity [31]	classic, tall cell, columnar cell hobnail Warthin-like solid microcarcinoma
	iso- or hypoechogenicity [31] smooth or spiculated/microlobulated margins [31] no microcalcifications [31] possible macrocalcifications [31] non-parallel orientation [31] mixed vascularity [31]	Hürthle cell
*BRAF^K601E^*	hypoechogenicity [31,39] spiculated/microlobulated margins [31,39] microcalcifications (20%) [31,39]	infiltrative FV PTC
*RAS*	hypoechogenicity [31] smooth margins [31] no microcalcifications [31] ovoid-to-round shape [29] mixed vascularization [29] non-parallel orientation [29,39]	invasive EFV PTC
	iso-/hyperechogenicity [46,50,51] well-defined margins [46,50,51] no calcifications [46,50,51]	NIFTP
*RET/PTC* rearrangement	diffuse involvement of one or both thyroid lobes [12,37] scattered microcalcifications [12,37] heterogeneous, mostly hypoechoic [12] ill-defined margins [12,37] nodule may not be defined (a “snowstorm” pattern) [12] hypo-/isoechogenicity [12,37] lymph node involvement with nodal microcalcifications [12,37]	diffuse sclerosing
	small nodule size [19] hypoechogenicity [19] microcalcifications [19] ill-defined margins [19]	classic
*PAX8-PPARγ*	hypoechogenicity [31] smooth margins [31] no calcifications [31] ovoid-to-round shape [31] mixed vascularization [31]	invasive EFV PTC
*CTNNB1* *APC*	multiple tumors [12,65,66] solid structure [12,65,66] hypoechogenicity [12,65] oval shape [12,65] well circumscribed [12,65] absence of hypoechoic halo [12] absence of calcifications [12,65]	cribriform-morular
*TERT* promoter	hypoechogenicity [12,33] microcalcifications [12,33] non-parallel orientation [60] microlobulated margins [60]	cribriform-morular hobnail classic

## Supplementary Materials

The following are available online at https://www.mdpi.com/2077-0383/8/11/1916/s1, Figure S1: Study selection flowchart outlining the protocol adopted in this review based on the Preferred Reporting Items for Systematic Reviews and Meta-Analyses (PRISMA) Four-Phase Flow Diagram.

## Abbreviations

ACR	American College of Radiology
ATA	American Thyroid Association
DSV	Diffuse sclerosing variant
EFV	Encapsulated follicular variant
ETA	European Thyroid Association
FAP	Familial adenomatous polyposis
FNAB	Fine needle aspiration biopsy
FV	Follicular variant
NIFTP	Non-invasive follicular thyroid neoplasm with papillary-like nuclear features
PTC	Papillary thyroid carcinoma
US	Ultrasound
WV	Warthin-like variant
