# Supplementary material for: Correlations between Molecular Landscape and Sonographic Image of Different Variants of Papillary Thyroid Carcinoma
Source: J Clin Med. 2019 Nov 8;8(11):1916. doi: 10.3390/jcm8111916 (PMC6912205; doi:10.3390/jcm8111916)
Supplement: Supplementary file 1 [file jcm-08-01916-s001.pdf]

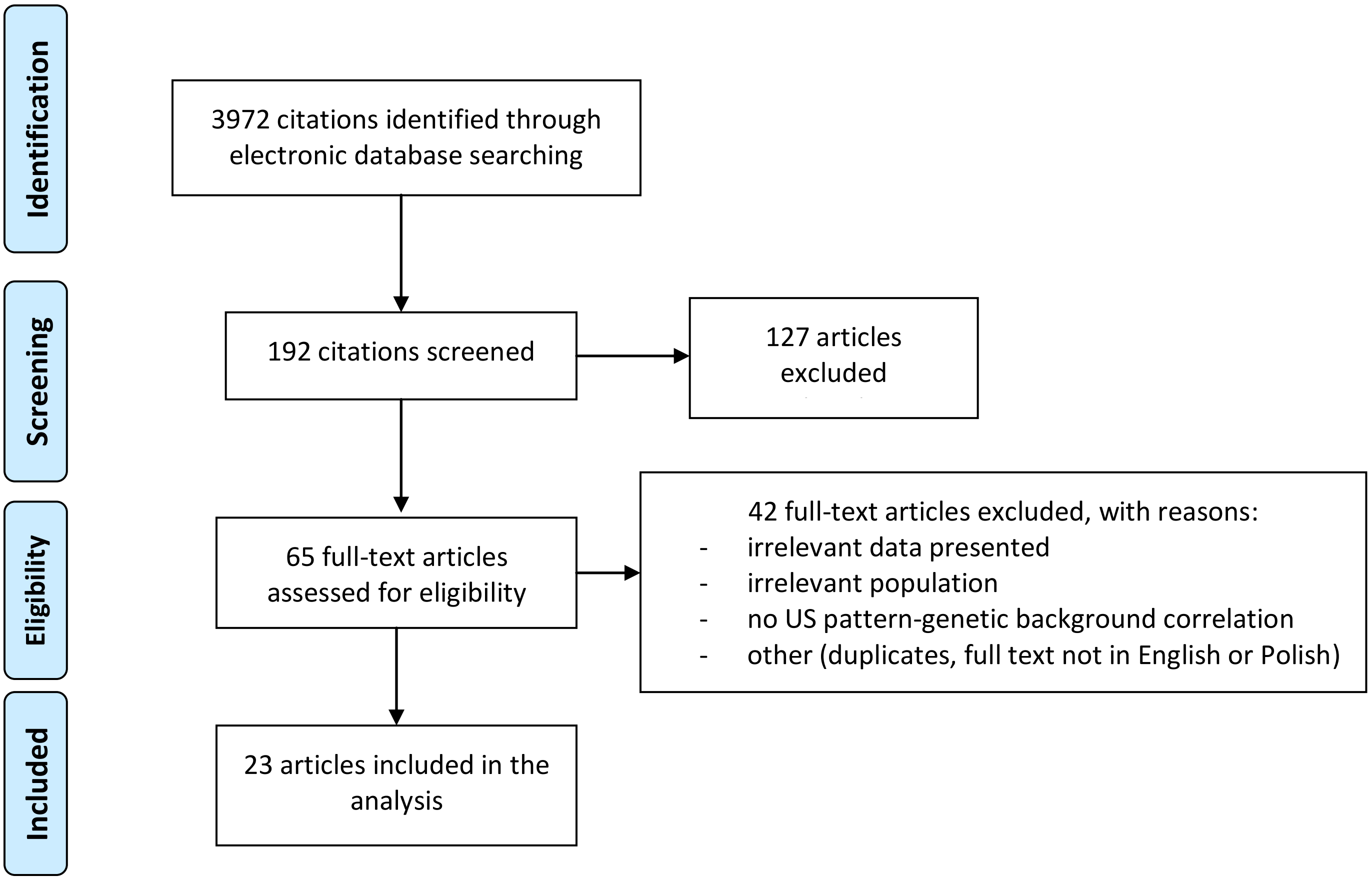

Figure S1. Study selection flowchart outlining the protocol adopted in this review based on the Preferred Reporting Items for Systematic Reviews and Meta-Analyses (PRISMA) Four-Phase Flow Diagram.
